# Dietary Polyphenols and Their Role in Oxidative Stress-Induced Human Diseases: Insights Into Protective Effects, Antioxidant Potentials and Mechanism(s) of Action

**DOI:** 10.3389/fphar.2022.806470

**Published:** 2022-02-14

**Authors:** Mithun Rudrapal, Shubham J. Khairnar, Johra Khan, Abdulaziz Bin Dukhyil, Mohammad Azam Ansari, Mohammad N. Alomary, Fahad M. Alshabrmi, Santwana Palai, Prashanta Kumar Deb, Rajlakshmi Devi

**Affiliations:** ^1^ Department of Pharmaceutical Chemistry, Rasiklal M. Dhariwal Institute of Pharmaceutical Education and Research, Pune, India; ^2^ Department of Pharmacology, MET Institute of Pharmacy, Nashik, India; ^3^ Department of Medical Laboratory Sciences, College of Applied Medical Sciences, Majmaah University, Al Majmaah, Saudi Arabia; ^4^ Health and Basic Sciences Research Center, Majmaah University, Al Majmaah, Saudi Arabia; ^5^ Department of Epidemic Disease Research, Institute for Research and Medical Consultations (IRMC), Imam Abdulrahman Bin Faisal University, Dammam, Saudi Arabia; ^6^ National Centre for Biotechnology, King Abdulaziz City for Science and Technology (KACST), Riyadh, Saudi Arabia; ^7^ Department of Medical Laboratories, College of Applied Medical Sciences, Qassim University, Buraydah, Saudi Arabia; ^8^ Department of Veterinary Pharmacology and Toxicology, College of Veterinary Science and Animal Husbandry, OUAT, Bhubaneswar, India; ^9^ Life Sciences Division, Institute of Advanced Study in Science and Technology, Guwahati, India

**Keywords:** dietary polyphenols, flavonoids, oxidative Stress, antioxidant, biomarkers, cellular signaling, protective function, mechanism of action

## Abstract

Dietary polyphenols including phenolic acids, flavonoids, catechins, tannins, lignans, stilbenes, and anthocyanidins are widely found in grains, cereals, pulses, vegetables, spices, fruits, chocolates, and beverages like fruit juices, tea, coffee and wine. In recent years, dietary polyphenols have gained significant interest among researchers due to their potential chemopreventive/protective functions in the maintenance of human health and diseases. It is believed that dietary polyphenols/flavonoids exert powerful antioxidant action for protection against reactive oxygen species (ROS)/cellular oxidative stress (OS) towards the prevention of OS-related pathological conditions or diseases. Pre-clinical and clinical evidence strongly suggest that long term consumption of diets rich in polyphenols offer protection against the development of various chronic diseases such as neurodegenerative diseases, cardiovascular diseases (CVDs), cancer, diabetes, inflammatory disorders and infectious illness. Increased intake of foods containing polyphenols (for example, quercetin, epigallocatechin-3-gallate, resveratrol, cyanidin etc.) has been claimed to reduce the extent of a majority of chronic oxidative cellular damage, DNA damage, tissue inflammations, viral/bacterial infections, and neurodegenerative diseases. It has been suggested that the antioxidant activity of dietary polyphenols plays a pivotal role in the prevention of OS-induced human diseases. In this narrative review, the biological/pharmacological significance of dietary polyphenols in the prevention of and/or protection against OS-induced major human diseases such as cancers, neurodegenerative diseases, CVDs, diabetes mellitus, cancer, inflammatory disorders and infectious diseases have been delineated. This review specifically focuses a current understanding on the dietary sources of polyphenols and their protective effects including mechanisms of action against various major human diseases.

## Introduction

Dietary polyphenols comprise a significant group of naturally occurring phytochemicals which primarily include phenolic acids, flavonoids, catechins, tannins, lignans, stilbenes and anthocyanidins. They possess antioxidant, chemopreventive and a wide range of pharmacological properties (Khan et al., 2021). Basically, our diet includes grains, cereals, pulses, vegetables, spices, fruits, chocolates, and beverages like fruit juices, tea, coffee and wine. They are rich in polyphenolic compounds of medicinal importance. Over 8,000 polyphenols have been reported from plants, out of several hundreds of polyphenols exist in human diets (Arts and Hollman, 2005). Research and clinical studies suggest that dietary polyphenolic compounds are linked to the maintenance of human health and prevention of diseases (Pandey and Rizvi, 2009). Dietary Polyphenols can effectively lower the risk of developing a wide range of human ailments such as cancer, cardiovascular diseases (CVDs), diabetes, inflammatory diseases and neurodegenerative disorders, just to name a few (Pandey and Rizvi, 2010).

Organic compounds bearing an aromatic ring with at least one hydroxyl group are termed as “phenolics”. In case, a compound possesses one or more aromatic rings having more than one hydroxyl group are called polyphenols (or polyphenolic compounds). Phenolics in plant derived foods are basically divided into phenolic acids, flavonoids, and non-flavonoids (Tsao, 2010). Phenolic acids are composed of hydroxyl by-products of aromatic carboxylic acids bearing a single phenolic ring. As per the C1-C6 or C3-C6 backbone, they are usually referred to as derivatives of benzoic acid or cinnamic acid. Flavonoids being the dominant class of plant polyphenols consist of two phenolic rings connected by a three-carbon bridge with a common C6-C3-C6 structural skeleton (Rudrapal and Chetia, 2017).

Oxidative stress (OS) is considered either a primary or a secondary cause for many chronic inflammatory diseases, neurodegenerative illness, metabolic disorders, cancer and CVDs. Dietary intake of fresh fruits and vegetables have clear effects against a number of diseases that involve OS. However, the role of the dietary polyphenols of their antioxidant abilities is still unclear. Dietary polyphenols (or flavonoids) act as efficient free radicals and reactive oxygen species (ROS) scavengers (according to biochemical scavenger theory) owing to the presence of aromatic structural feature, multiple hydroxyl groups, and a highly conjugated system (Salisbury and Bronas, 2015). They have the capability to negate ROS or to suppress cellular OS enabling them to avert oxidative damages of biomolecules (lipids, proteins, DNA) and thereby diminish tissue inflammation (Zhang and Tsao, 2016). This is referred to as antioxidant effects of dietary polyphenols. The ability of dietary polyphenols to suppress inflammation and consequently oxidative damage to tissues is mediated through their antioxidant effects, interference with signaling pathways of OS and suppression of signaling transduction mechanism of pro-inflammatory mediators and cellular inflammatory pathways at molecular level (Apel and Hirt, 2004).

Pre-clinical and clinical evidence strongly suggest that long term consumption of diets rich in polyphenols offer protection against the development of various chronic diseases such as neurodegenerative diseases, cardiovascular diseases (CVDs), cancer, diabetes, inflammatory disorders and infectious illness (Khan et al., 2021). Increased intake of foods containing polyphenols (for example, quercetin, epigallocatechin-3-gallate, resveratrol, cyanidin etc.) has been claimed to lower the incidence of a majority of chronic oxidative cellular damage, DNA damage, tissue inflammations, various cancers, viral/bacterial infections, and neurodegenerative diseases (Pandey and Rizvi, 2009; Shahidi and Ambigaipalan, 2015; Egbuna et al., 2021; Khan et al., 2021).

In this narrative review, the biological/pharmacological significance of dietary polyphenols in the prevention of and/or protection against OS-induced major human diseases such as cancers, neurodegenerative diseases, CVDs, diabetes mellitus, cancer, inflammatory disorders and infectious diseases have been delineated. This review specifically focuses a current understanding on the dietary sources of polyphenols and their protective effects including mechanisms of action against various major human diseases.

## Oxidative Stress and Its Role in Disease Pathogenesis

The bulk of free radicals that causes damage to biological structures (i.e., biomolecules such as proteins, lipids, DNA) are oxygen-free radicals, also known as reactive oxygen species (ROS). ROS include superoxide anion radical (O_2_•^–^), perhydroxyl radical (HOO•), nitric oxide radical (NO•), hydrogen peroxide (H_2_O_2_), singlet oxygen (^1^O_2_), hydroxyl radical (•OH), hypochlorous acid (HOCl), hypochlorite radical (ClO^−^), peroxynitrite (ONOO^−^), and lipid peroxides (LOPs). ROS may be generated from various exogenous sources such as UV light irradiation, X-rays, γ rays, metal catalyzed reactions, environmental carcinogens/toxins. Heavy/transition metals, alcohol, tobacco, synthetic solvents, drugs (e.g., tacrolimus, cyclosporine, bleomycin, and gentamycin), culinary sources (e.g., waste oil, fat and smoked meat), and radiation are all exogenous sources of ROS. Endogenous sources of ROS include cytochrome P450 metabolism, mitochondrial reactions, peroxisomes, and inflammatory cell activation. Whether endogenous or exogenous, ROS when increased or excessively produced can cause oxidative changes/damages to all cellular macromolecules. Excessive intracellular production of ROS builds up cellular OS that usually cause damage to lipids, proteins, DNA and carbohydrates. Thus, OS has been linked to the pathogenesis of many human diseases including brain dysfunction, cancer, inflammatory diseases, heart diseases, diabetes and many others (Law et al., 2017).

**FIGURE 1:**
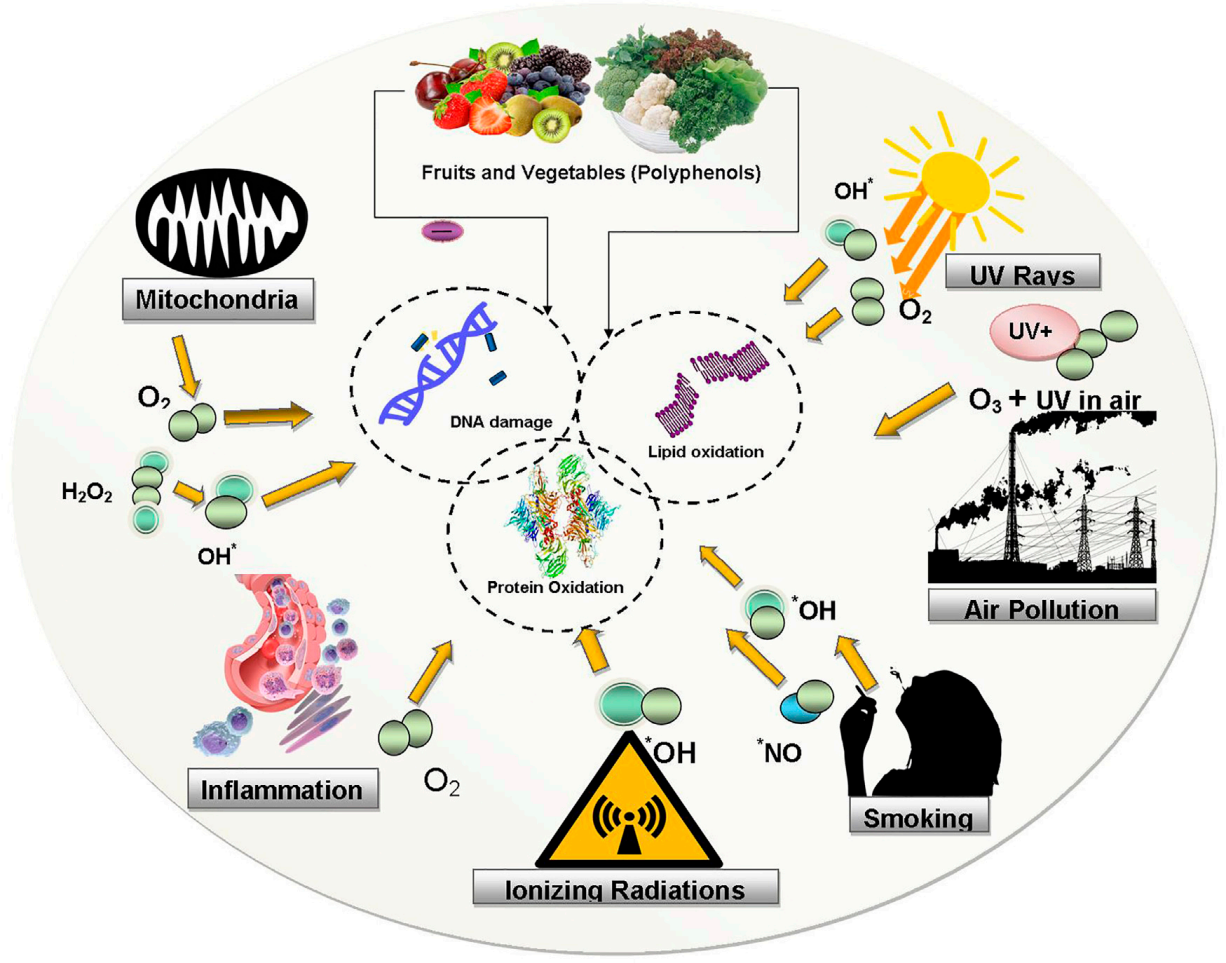
Deleterious effects of OS/ROS on biomolecules. ROS generated from various sources (environmental/biological) cause oxidations of lipid, protein and DNA molecules. Abbreviations: UV: ultraviolet rays, OH: hydroxyl, NO: nitrogen oxide, O2: oxygen, O3: Ozone, H2O2: hydrogen peroxide.

Human body has its own in-built biological process/mechanism to defend itself against foreign threats and pathogenic microorganisms, including natural antioxidant defense, immunity and/or DNA repair enzymes. Several antioxidant enzymes such as superoxide dismutase (SOD), catalase (CAT), and reduced glutathione (GSH) aid in the removal of free radicals (Halliwell and Gutteridge, 2015). When not well managed, OS causes extensive chronic and degenerative diseases, the aging process, and acute pathologies like trauma and stroke. Most importantly, when the production of free radicals overwhelms the antioxidant defenses, it leads to OS, the harmful mechanism that can significantly change cell membranes and other biological structures such as lipoproteins, lipids, proteins, DNA etc. (Genestra, 2007). Figure 1 depicts deleterious effects of OS/ROS on biomolecules.

Membrane lipids are vulnerable to peroxidative reactions. Hydroxyl radical (•OH) is an essential reactive moiety and originator of the ROS chain reaction in polyunsaturated lipoperoxidation process. Several compounds are formed as a result of lipid polysunsaturated fatty acids (PUFA) peroxidation, namely isoprostanes, malondialdehyde (MDA), 4-hydroxy-2-nonenal (4-HNE) etc. (Lobo et al., 2010; Kunwar and Priyadarsini, 2011). These compounds are used as biomarkers in lipid peroxidation assays and have been linked to neurodegenerative diseases, heart disease, and diabetes (Genestra, 2007; Lü et al., 2010; Pandey and Rizvi, 2010). Peroxynitrite can also destroy lipoproteins and causes lipid peroxidation of cell membranes. ROS can also affect protein synthesis and protein functions. Protein oxidation can result in amino acid modifications (oxidative protein modification), accumulation of cross-linked reaction products, peptide chain fragmentation, and augmented electrical charges (Parthasarathy et al., 1999; Krishnamurthy and Wadhwani, 2012). Chemical agents that generate oxygen-free radicals like ionizing radiations and activated oxygen cause DNA damage which results in mutations, deletion, and similar lethal genetic effects. Oxidative DNA damage causes the development of various oxidative DNA lesions, which may trigger mutations (Halliwell and Gutteridge, 2015). Because of DNA disruption, base moieties and sugar become more vulnerable to oxidation, resulting in protein cross-linking, base degradation, and single-strand breakage (Zadák et al., 2009). Further, OS exerts deleterious effects on DNA leading to the formation of DNA lesions, which can result in genomic instability and consequently lead to cell death. The guanine (a base of DNA) is most susceptible to oxidation in cellular OS. In the presence of ROS, the oxidation of guanosine to 8-oxoguanosine (8-oxoG) takes place. The formation of 8-oxoG is the most common lesion in the DNA molecule. When 8-oxoG is inserted during DNA replication, it could generate double-strand breaks, which finally causes damage to DNA molecule (Aguiar et al., 2013).

**FIGURE 2:**
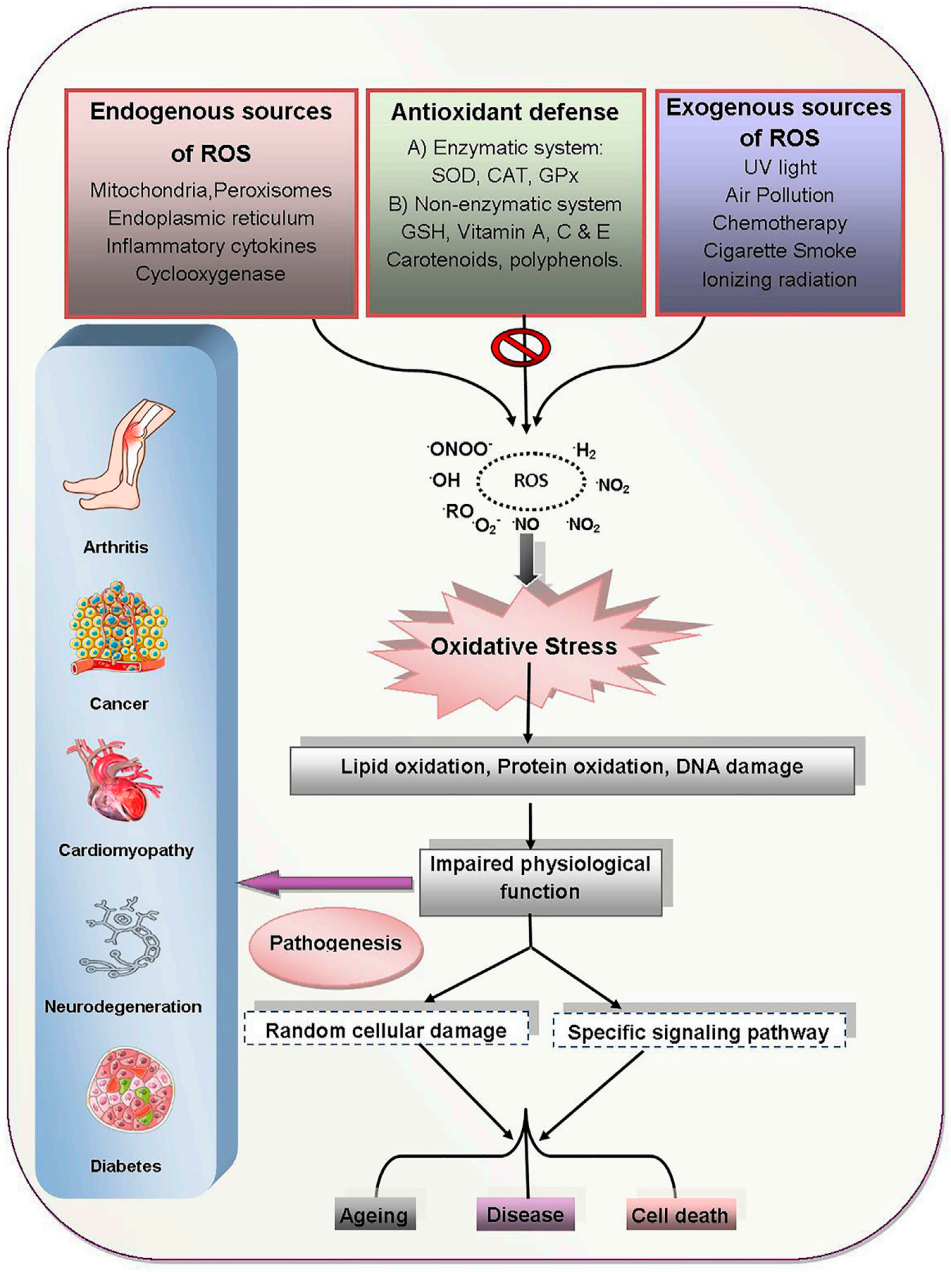
OS induced human diseases and their pathogenesis. ROS generated from exogenous/endogenous sources induce OS which results in the pathogenesis of various human diseases through impaired physiological functions, cellular damages and specific signaling dysfunctions. Abbreviations: ROO: alkoxyl radical, ROO: peroxyl radical, ONOO^−^: perooxynitrite, NO_2_^•^: nitrogen dioxide radical, O_2_^•−^: superoxide radical, ROS: reactive oxygen species, SOD: superoxide dismutase, CAT: catalase, GPx: glutathione peroxidase, GSH: glutathione.

Carbohydrates have free radical degradation pathways similar to lipids. The development of oxygen-free radicals throughout initial glycation can lead to glycoxidative harm to biological tissues (Benov and Beema, 2003). During the glycoxidation process, many reactive aldehydes, including 4-HNE and MDA are formed resulting in advanced glycation termination products (Phaniendra et al., 2015). The pathophysiological changes that take place during OS induced diseases are outlined in Figure 2.

## Dietary Polyphenols, Their Chemistry and Sources

Polyphenols are found naturally in fruits and vegetables such as cereals, pulses, dried legumes, spinach, tomatoes, beans, nuts, peppermint, cinnamon, pears, cherries, oranges, apples, red wine, tea, cocoa, coffee and so on (Arts and Hollman, 2005; Scalbert et al., 2005). Polyphenols are classified into different groups depending on the number of aromatic (phenolic) rings they contain and the structural elements that connect these rings. They are broadly grouped into phenolic acids, flavonoids, stilbenes and lignans (Khan et al., 2021). Plant derived polyphenolic compounds (for example, phenolic acids and flavonoids) occurs in conjugated forms with one or more sugar residues (as glycosides) bound to hydroxyl groups through direct linkages of the polysaccharide or monosaccharide-like sugar to an aromatic carbon (Rudrapal and Chetia, 2017). It is naturally bound to a variety of other molecules, including carboxylic and organic acids, lipids, amines, and other phenolic compounds (Kondratyuk and Pezzuto, 2004).

**FIGURE 3:**
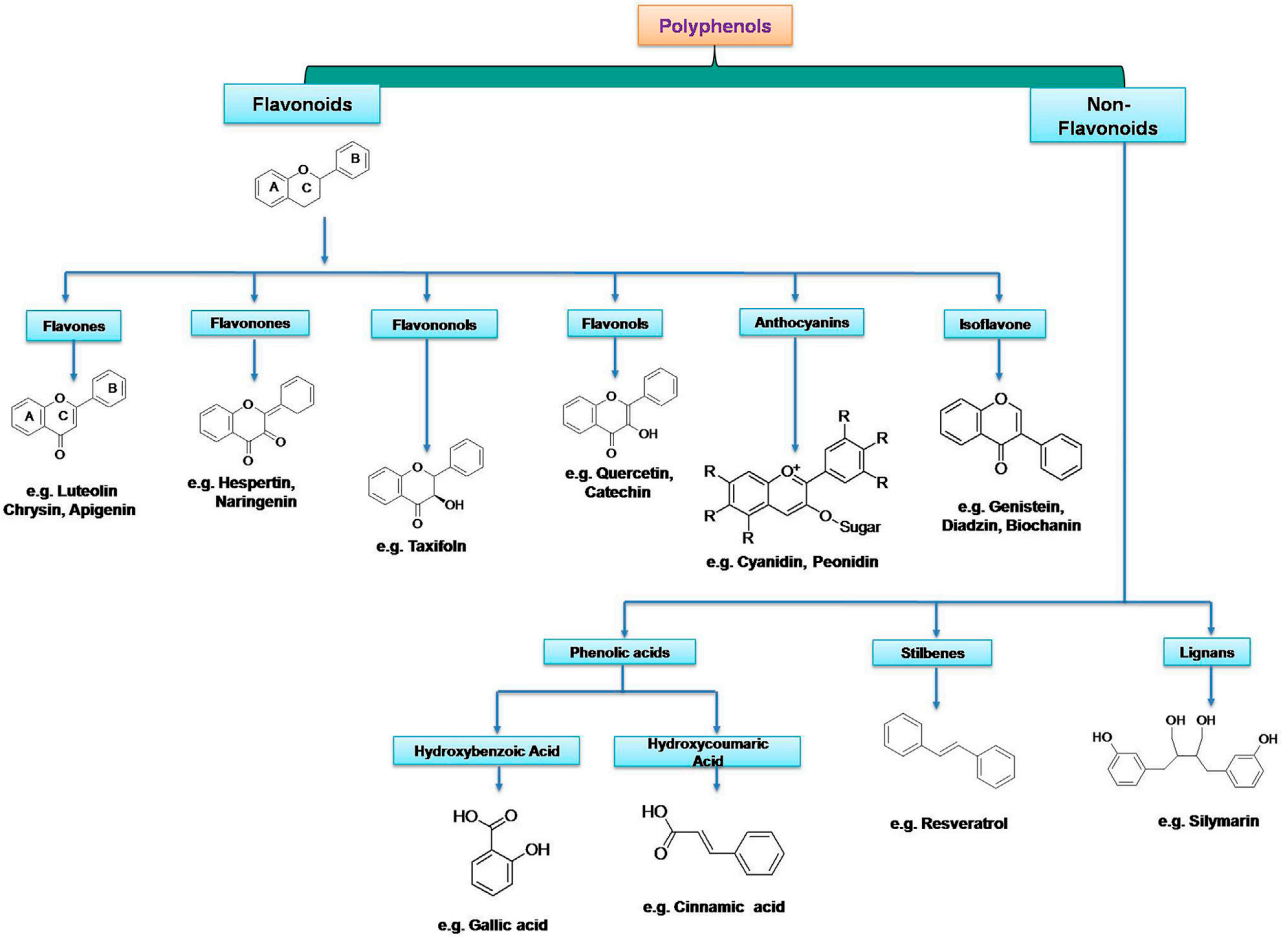
Different classes of plant polyphenols with their basic structural scaffolds. Structural scaffolds represent the chemistry behind various classes of polyphenolic substances.

**FIGURE 4:**
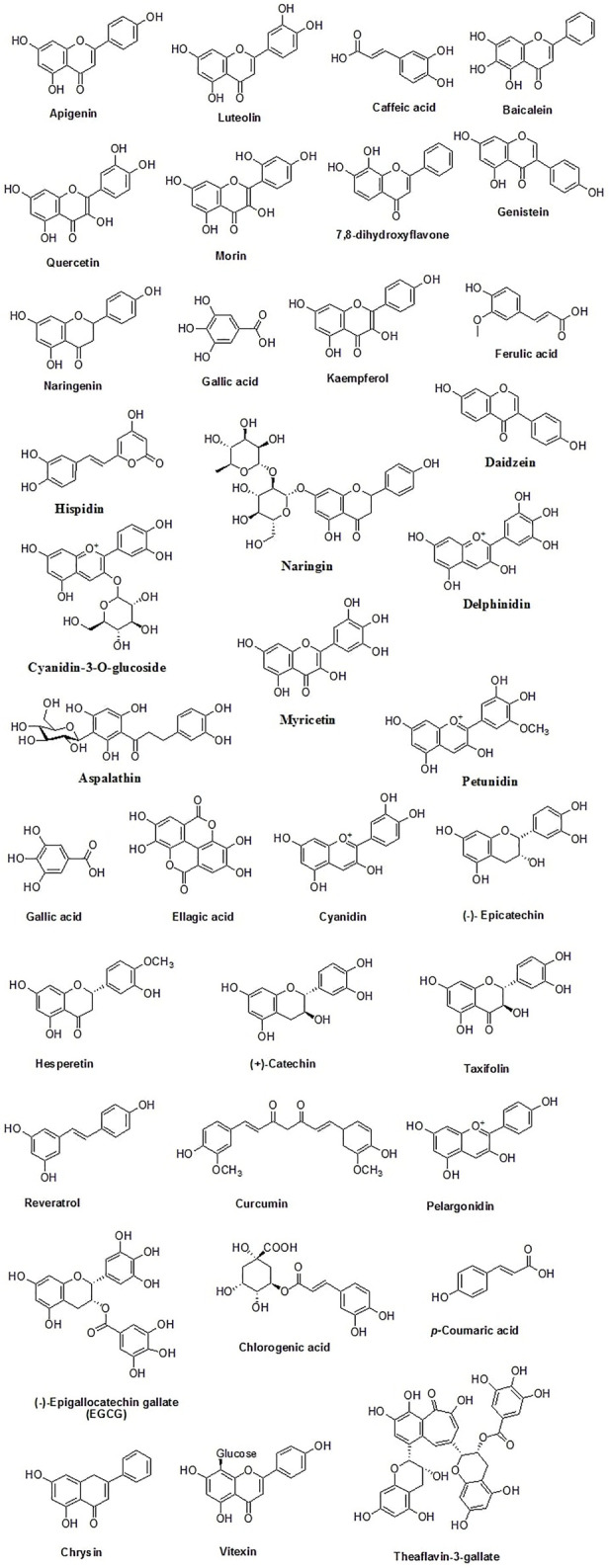
Chemical structures of some common dietary polyphenols of medicinal importance.

Dietary polyphenolics can be broadly classified into flavonoids and other polyphenols (non-flavonoids). Flavonoids are further classified into different subgroups based on their structures such as flavan-3-ols (examples: catechin, epicatechin, epigallocatechin), isoflavones (examples: genistein, genistin, daidzenin, daidzin, biochanin A, formononetin), flavones (examples: luteolin, apigenin, chrysin), flavonones (examples: hesperetin, naringenin), flavonols (examples: quercetin, kaempferol, galangin, fisetin, myricetin), flavononol (example: taxifolin), flavylium salts (examples: cyanidin, cyanin, pelargonidin), and flavanones (examples: hesperetin, naringenin, eriodictyol, isosakuranetin) (Pietta, 2000; Barreca et al., 2017). Non-flavonoid polyphenols can be further classified into phenolic acids (examples: cinnamic acid, *p*-coumaric acid, caffeic acid, ferulic acid, sinapic acid, gentisic acid, vanillic acid, gallic acid, syringic acid, protocatechuic acid), tannins (examples: procyanidins, catechin, afzelechin, gallocatechin, ellagic acid, gallic acid gallate, gallotannin, ellagitannin, hexahydroxydiphenic acid), lignans (examples: niranthin, sesamin, silymarin, rubrifloralignan A, bicyclol, phillygenin, clemastanin B, isatindolignanoside A, diphyllin, hinokinin, yatein, secoisolariciresinol etc.), anthocyanidins (examples: cyanidin, delphinidin, pelargonidin, peonidin, petunidin, and malvidin etc.), anthraquinones (examples: physcion, chrysophanol, aloe-emodin, rhein etc.), coumarins (examples: osthole, anthogenol, ammoresinol, phellodenol etc.), and stilbenes (examples: resveratrol, piceatannol, rhapontigenin, isorhapontigenin, pinosylvin, pterostilbene etc.) (Serrano et al., 2009; Reinisalo et al., 2015; Abotaleb et al., 2020; Cui et al., 2020; Luca et al., 2020). Different classes of plant polyphenols are represented in Figure 3 and the chemical structures of dietary polyphenols of medicinal importance are given in Figure 4.

In plant derived polyphenolic compounds, flavonoids comprise the largest group with an approximately 10,000 natural analogues. They are hydroxylated aromatic compounds often exist as bright coloured (yellow to red) pigments in the plants and microbes (Cook and Samman, 1996). The structural framework of flavanoid compounds comprises benzo-γ-pyrone ring system (C6-C3-C6 backbone). Structurally, they are characterized as C15 compounds and composed of two phenolic (C6) rings which are linked by a bridge of heterocyclic pyrone rings. Two phenolic rings are denoted as A and B rings, whereas, connecting heterocyclic rings is considered as C ring in the structural skeleton (Cook and Samman, 1996; Tresserra-Rimbau et al., 2018).

Phenolic acids are dominant category under the non-flavonoid class of polyphenols and further subdivided into hydroxybenzoic acids (C1-C6 backbone) and hydroxycinnamic acids (C3-C6 backbone) and structurally characterized by a carboxylic acid group linked to the phenolic ring (Durazzo et al., 2019). They generally exist in the plants either in free form or esterified form. They also exist as a conjugate with sugar moiety and proteins often and hydrolysable on acid or alkali treatment. Many foods and beverages like wine, tea, coffee chocolate, vegetables, whole grains and fruits contain hydroxycinnamic acid in very high concentrations (Tsao, 2010; Panche et al., 2016).

Stilbenes are biosynthesized by plants during external influence such as infection or injury. They contain C6-C2-C6 backbone and structurally represent 1,2-diphenylethylene nucleus and exist either in the monomeric or oligomeric form. Resveratrol is a naturally occurring important bioactive compound that comes under this category (Tresserra-Rimbau et al., 2018; Liu et al., 2019).

Like stilbenes, a coumarin type of polyphenols, also synthesize and accumulate in the plant tissues due to the abiotic stress and microbial attacks. They are composed of 1,2-benzopyrone skeleton (α-chromone). They also frequently exist in the prenylated form. Coumarin cores are often used as a template in the synthesis of various pharmacologically important novel compounds (Shen et al., 2009; Tresserra-Rimbau et al., 2018).

Lignans are a comparatively less abundant class of phenolic compounds structurally characterized by a dibenzylbutane skeleton. These types of compounds are generally found in higher plants (gymnosperms, angiosperms, pteridophytes etc.). Often they are found in the plant material in bound form and make difficulty in extraction (Shen et al., 2009; Tresserra-Rimbau et al., 2018).

Anthocyanidins are the bright coloured (blue, red, or purple pigments) flavonoid compounds found in the flowers, fruits and leaves etc. These are positively charged compounds containing flavylium cations and often occur as chloride salts (Shen et al., 2009). Anthocyains are composed of one or more sugar moieties in the C-3 position of the C ring. Frequently these compounds are found in the plants as a conjugate with phenolic acids and other organic acids. The de-glycosylated forms of anthocyanins are called anthocyanidins. Variation in the colour of the anthocyanin compounds is reliant to the pH acylation and methylation -OH groups attached to the A and B ring and also pH of the environment (Khoo et al., 2017).

Proanthocyanidins are the dimer or trimer of flavanols in condensed form, also known as condensed tannins. Based on the interflavanic linkages, they can be divided as type A (C2–*O*–C7 or C2–*O*–C5 bonding), or type-B (C4–C6 or C4–C8). They often produced from flavanol rich materials during fermentation (Khoo et al., 2017). Open C rings containing flavanoids are categorized as chalcones. Chalcone compounds exerts a common chemical scaffold of 1,3- diaryl-2-propen-1-one which is also known as chalconoid (Zhuang et al., 2017).

Dietary polyphenolics are most abundantly found in seasonings (examples: cloves, dried peppermint, star anise, celery seed, rosemary, spearmint, ginger, ceylan cinnamon, parsley, marjoram, vinegar), cocoa products (examples: cocoa powder, dark chocolate, milk chocolate), fruits (examples: black chokeberry, black elderberry, lowbush blueberry, blackcurrant, highbush blueberry, plum, sweet cheery, blackberry, strawberry, red raspberry, prune, black grape, apple, peach, redcurrant, apricot, nectarine, quince, pear, green grape), seeds (examples: flaxseed, chestnut, hazelnut, pecan nut, soy flour, roasted soyabean, almond, soy, black bean), vegetables (examples: black olive, green olive, globe artichoke heads, red chicory, red onion, green chicory, spinach, shallot, yellow onion), cereals (examples: whole grain hard wheat flour, refined maize flour, whole grain rye flour, whole grain wheat flour, whole grain oat flour), alcoholic beverages (examples: red wine, white wine, rose wine), non-alcoholic beverages (examples: coffee, black tea, green tea, pure apple juice, pure pomegranate juice, pure blood orange juice, pure grapefruit juice, pure lemon juice, chocolate beverage with milk, soy milk, pure pummelo juice) and oils (examples: extra-virgin olive oil, rapeseed oil) (Pandey and Rizvi, 2009; Pérez-Jiménez et al., 2010; Reinisalo et al., 2015).

## Polyphenols and Their Protective Effects Against Human Diseases

### Aging and Neurodegenerative Disorders

Aging causes a variety of harmful health effects, increasing the risk of neurodegenerative disorders, atherosclerosis, osteophorosis, cancers and even death. The free radical theory of aging (also known as OS theory) is well accepted as the aging progresses. Although free radicals may be a key player in the aging process, they do not play any central role in that. Numerous cell-centric hypotheses has also been attributed in aging and related disorders (Tabibzadeh, 2021). Since the potential of antioxidative and repair pathways declines with age, oxidative damage to biological tissues rises (Rizvi and Maurya, 2007). In aging, the accumulation of ROS causes OS to brain biomolecules (proteins, DNA, and lipids) leading to progression of neurodegenerative diseases (Barnham et al., 2004). The most common neurodegenerative disease is Alzheimer’s disease (AD), which affects millions of people across the globe. Studies reveal that oxidative disruption plays a critical role in several major brain dysfunctions such as AD, Parkinson’s disease (PD), memory loss, amyotrophic lateral sclerosis (ALS), depression multiple sclerosis etc. (Pandey and Rizvi, 2010).

The consumption of antioxidant-rich diets decreases the harmful consequences of aging and neurodegenerative illness. Fruits and vegetables contain polyphenolic compounds with antioxidants and anti-inflammatory activities have been well reported to exhibit anti-aging properties in rats and mice (Joseph et al., 2005). Anthocyanins found in abundance in bright colored fruits such as berry fruits, tomatoes, oranges etc. have strong antioxidant and anti-inflammatory properties, inhibiting lipid peroxidation as well as cyclo-oxygenase (COX-1 and COX-2) pathways (Reis et al., 2016). Dietary supplements containing elevated amounts of flavonoids from strawberries, lettuce, or blueberries aid in the reversal of age-related discrepancies in the brain and behavioral control in aged rats (Shukitt-Hale et al., 2008). Tea catechins have antioxidant properties that might be associated with anti-aging. The *in vitro* effect of tea catechins on erythrocyte malondialdehyde (MDA), reduced glutathione (GSH), and on membrane sulphydryl (-SH) group in humans has been reported by Maurya and Rizvi (2009). Polyphenols can also help to reduce the negative effects of aging on the brain and nervous system. EGCG reduces the progression of ALS (in a mouse model), which is crucial for their significance in the protection of the aging of brain (Xu et al., 2006). Resveratrol, a polyphenol found in grapes and red wine, has anti-aging property.

**FIGURE 5:**
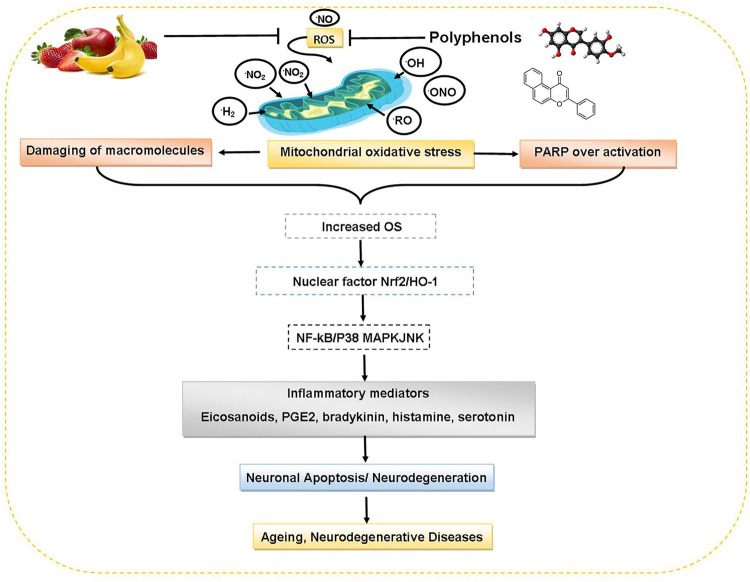
Protective roles of dietary polyphenols against aging and neurodegenerative disorders. Abbreviations: Nrf 2: nuclear factor erythroid 2, HO-1: heme oxygenase-1, NF-kB: nuclear factor kappa-light-chain-enhancer of activated B cells, P38 MAPK: protein 38 mitogen-activated protein kinase, JNK: Jun N-terminal kinase, PGE2: prostaglandin E2.

Fruits and vegetables rich in polyphenols are potential neuroprotective agents which can modulate many cellular processes like apoptosis, redox balance signaling, differentiation and proliferation. Polyphenols being antioxidative agents can protect against various neurological diseases. Resveratrol shows neuroprotective effect against models of AD (Rahman et al., 2020). Resveratrol hunts O^2−^• and OH^−^• free radicals and lipid hydroperoxyl free radicals. Epigallocatechin gallate (EGCG) protects against the neurotoxin MPTP (N-methyl-4-phenyl-1,2,3,6-tetrahydropyridine) which can induce Parkinson’s-like disease, through competitively inhibition of drug absorption or by scavenging MPTP-mediated radical formation (Rossi et al., 2008). Figure 5 delineates the protective roles of dietary polyphenols against aging and neurodegenerative disorders.

### Cardiovascular Diseases

**FIGURE 6:**
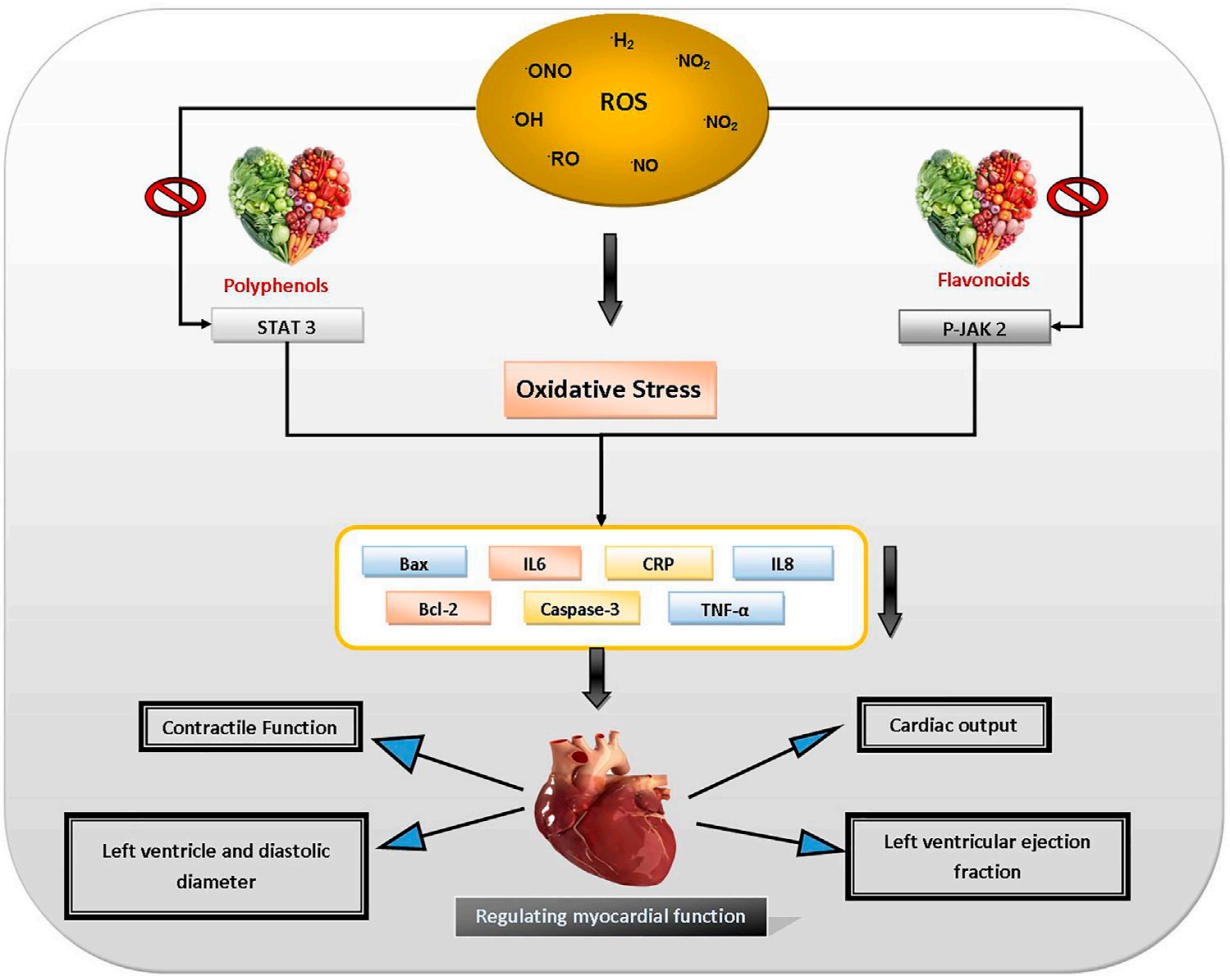
Protective effects of dietary polyphenols against CVDs. Abbreviations: Bax: BCL2 associated X apoptosis regulator, IL6: interleukin 6, CRP: C-reactive protein, IL8: interleukin 8, Bcl-2: B-cell lymphoma 2, Caspase-3: cysteine-aspartic acid protease 3, TNF-alpha: tumour necrosis factor - alpha, P-JAK 2: protein Janus kinase 2, STAT 3: signal transducer and activator of transcription 3

OS can be the primary or secondary reason for various CVDs. Preclinical evidence support that OS is linked to a variety of CVDs, including atherosclerosis, ischemia, stroke, cardiomyopathy, cardiac hypertrophy, and hypertension, as well as congestive heart failure (CHF) (Vita, 2005; Bahoran et al., 2007; Ceriello, 2008; Bonnefont-Rousselot, 2016). Consumption of polyphenol-rich foods reduces risk of CVDs (Khan et al., 2021). Recent studies indicate that polyphenols also exert beneficial effects on vascular disorders by blocking platelet aggregation as well as by preventing oxidation of low-density lipoprotein (LDL), ameliorating endothelial dysfunction, reducing blood pressure, improving antioxidant defenses and alleviating inflammatory responses. Polyphenols are powerful regulators of LDL oxidation, which is believed to be the main mechanism in the progression of atherosclerosis (Nardini et al., 2007). Polyphenols guard against CVDs because of their anti-inflammatory, antioxidant, antiplatelet effects, and also by increasing high-density lipoprotein (HDL) level. Dietary flavonoids may reduce endothelial disorders linked with various risk factors for atherosclerosis before plaque creation (Khan et al., 2021). Tea catechins suppress smooth muscle cell penetration and proliferation in the arterial wall (Bhardwaj and Khanna, 2013). Resveratrol inhibits platelet aggregation by selectively inhibiting cyclooxygenase 1 (COX-1), which augments production of thromboxane A2, platelet aggregation, and vasoconstrictor inducer (Senoner and Dichtl, 2019). It increases nitric oxide signaling in the endothelium, resulting in vasodilation (Harikumar and Aggarwal, 2008; Shi et al., 2019). Figure 6 depicts the protective effects of dietary polyphenols against CVDs.

### Diabetes Mellitus

**FIGURE 7:**
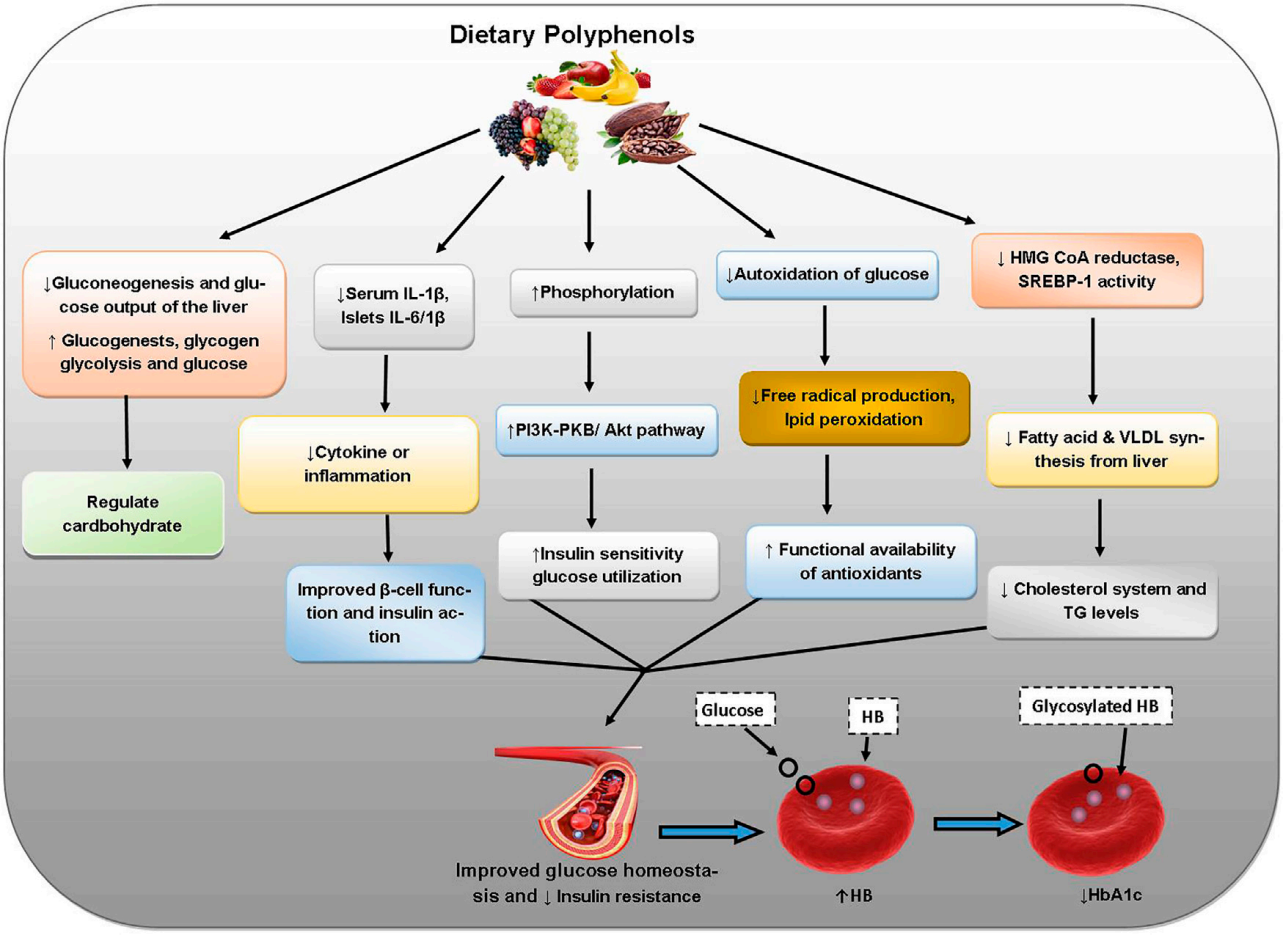
Protective roles of dietary polyphenols against diabetes. Abbreviations: IL-1 beta: interleukin-1β, IL-6/1B: interleukin-6, P13K-PKB: phosphoinositide-3-kinase–protein kinase B, Akt: AK strain transforming, HMG CoA reductase: β-hydroxy-β-methylglutaryl-CoA reductase, SREBP-1: sterol regulatory element-binding protein 1, VLDL: very low density lipoproteins, TG: Triglycerides, HB: Hemoglobin.

Abnormality in glucose metabolism leads to hyperglycemia and consequently diabetes mellitus (type-1 and type-2). Apart from co-morbidities like heart disease or stroke, chronic complications may develop in diabetes such as diabetic retinopathy affecting eyes cause blindness, nephropathy altered renal functions, and neuropathy causing nerve damage and numbness/paralysis (Rizvi and Zaid, 2001; Rizvi and Zaid 2005; Junejo et al., 2017; Junejo et al., 2018; Junejo et al., 2020a; Junejo et al., 2020b; Hussain et al., 2021; Junejo et al., 2021). Apigenin derivative possesses strong antidiabetic activity extending protection against the variations throughout OS in diabetes (Junejo et al., 2021). Quercetin decreases lipid peroxidation and inhibits cellular oxidation in diabetes (Pandey and Rizvi, 2009). Resveratrol prevents cytotoxicity and OS caused by excessive glucose levels. Resveratrol decreases diabetes-induced kidney alterations (diabetic nephropathy) and thereby increases renal disorder and OS in diabetic rats. Resveratrol reduces secretion of insulin and deferrers insulin resistance onset which may be due to the inhibition of K^+^ATP and K^+^ V channels in β cells (Chen et al., 2007; Oyenihi et al., 2016). The polyphenols of *Hibiscus sabdariffa* weaken diabetic nephropathy in terms of serum lipid profile and kidney oxidative markers (Lee et al., 2009). *H. sabdariffa* also contains flavonoids, protocatechuic acid, and anthocyanins. The ameliorating effects of a high antioxidant polyphenol supplement of green tea extract, pomegranate extract and ascorbic acid on OS due to type 2 diabetes have been proved through decreased LDL, reduced plasma MDA, and increased HDL indicating better antioxidant potential with augmented total plasma GSH with preventive action against cardiovascular complications as well (Fenercioglu et al., 2010). The flavonoid rutin also has antidiabetic effects (Ghorbani, 2017). Figure 7 outlines the protective effects of dietary polyphenols against diabetes mellitus.

### Cancer

The occurrence of cancer (or malignant diseases) is augmented with OS along with an increase in the amount of free radicals like ROS causing biomolecular (DNA) and tissue damages. ROS-induced DNA damage results in induction/replication errors, transcriptional arrest, and/or genomic instability allied with carcinogenesis (Federico et al., 2007; Khansari et al., 2009). Studies suggest that a diet that includes regular consumption of fruits and vegetables (rich in polyphenols such as catechins, resveratrol, ellagic acid, naringenin, quercetin etc.) significantly lowers the risk of developing many cancers. The chemopreventive action of polyphenols includes estrogenic and antiestrogenic involvement, antiproliferation, cell cycle arrest or apoptosis activation, oxidation resistance, induction of detoxification enzymes, host immune system regulation, anti-inflammatory activity, and improvements in cellular signaling (García-Lafuente et al., 2009). Polyphenols affect pro-carcinogen metabolism by moderating the cytochrome P450 enzymes expression involved in carcinogen stimulation (Talalay et al., 1988). Black tea polyphenols like EGCG, theaflavins and thearubigins have potent anticancer properties (Shankar, 2008; Sharma and Rao, 2009). Tea catechins with cancer prevention efficacy inhibit the conversion of intraepithelial prostate lesions to cancer. In prostate carcinoma cells, polyphenols from black tea suppress proliferation of increasing apoptosis (Kim et al., 2014). Anti-carcinogenic effects of resveratrol are due to the antioxidant function, which inhibits hydroperoxidase, Akt (PI3K-Akt) signaling pathway, matrix metalloprotease-9, NF-kB, protein kinase C, cyclooxygenase, focal adhesion kinase and Bcl-2 (B cell lymphoma 2) biomarkers/enzymes (Athar et al., 2007).

### Infectious Diseases

The emergence of multi-drug resistant (MDR) pathogens has become a global threat and a cause of significant morbidity and mortality around the world. Augmenting the OS pathway and induction of ROS formation has emerged as potential antimicrobial target in recent times. Flavonoids exhibit broad spectrum of antimicrobial actions through different mechanisms which are often observed little different than those of conventional antibiotics and thus could be of importance in the improvement of antimicrobial therapeutics (Dwyer et al., 2009; Rosillo et al., 2016). During bacterial infection, the host immune response leads to inflammation due to the generation of ROS, and consequently leading to OS. Increased OS may lead to the vulnerability of the infection and also triggers the malfunctioning of cellular metabolism (Kim et al., 2019). Flavonoids are well known for their modulatory effect against OS in the human body by scavenging free radicals and chelating the metallic ions (Ivanov et al., 2017; Tresserra-Rimbau et al., 2018). It is reported that many antibacterial drugs kill bacteria by activation of ROS pathways, whereas, a mild amount of ROS is proven to be beneficial to the microorganism for their signaling mechanisms. The therapeutic role of antioxidant polyphenols in mitigating OS-related tissue damage and inflammations in bacterial and viral infections is well defined. Black tea polyphenols have *in vitro* antiviral properties (Wu et al., 2015; Górniak et al., 2019). EGCG, the main constituent of polyphenol, has antiviral activities on a diverse range of viruses such as human immunodeficiency virus, influenza virus and hepatitis C virus (Steinmann et al., 2013). Polyphenolic compounds that have been reported in very preliminary *in silico* and *in vitro* studies to exhibit anti-SARS-CoV activity include quercetin, acacetin, apigenin, baicalein, hesperidin, morin, rutin, naringin, naringenin, (–)-catechin, (–)-catechin gallate, (–)**-**gallocatechin gallate, diosmin, daidzein, genistein, glycitein, kaempferol, luteolin, myricetin, silibinin, silymarin, orientin, curcumin, and oroxylin A (Sharma and Rao, 2009; Suzuki et al., 2016; Praditya et al., 2019; Vestergaard and Ingmer, 2019; Jennings and Parks, 2020; Gansukh et al., 2021).

### Inflammatory Diseases

**FIGURE 8:**
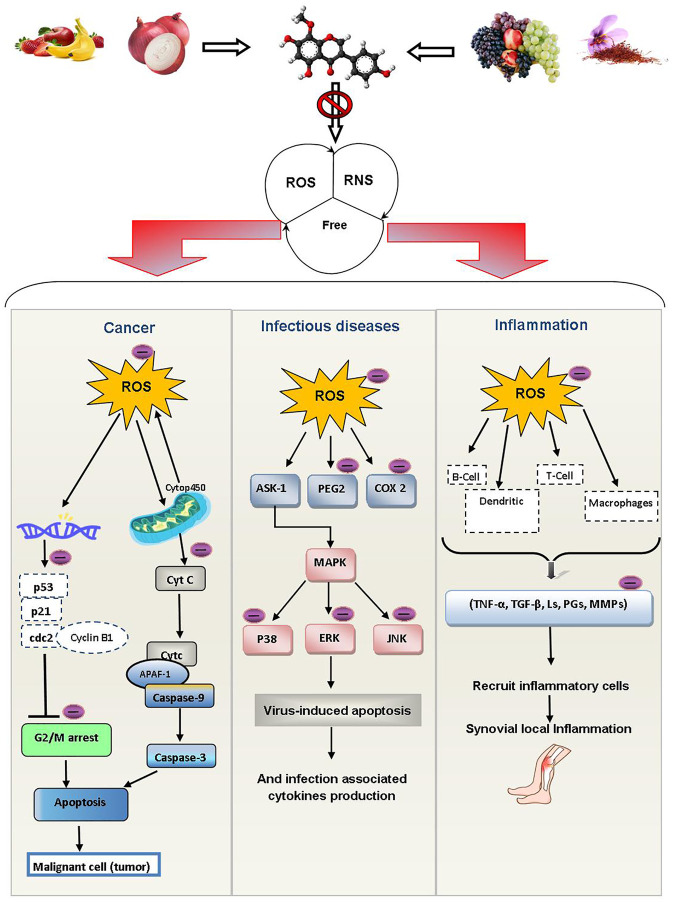
Protective effects of dietary polyphenols against cancer, infectious illness and inflammatory diseases. Abbreviations: ROS: reactive oxygen species, RNS: reactive nitrogen species, P53: tumor protein 53, P21: tumor protein 21, CDC2: cell division control 2, CASPASE-9: cysteine-dependent aspartate-directed protease-9, APAF-1: apoptotic protease activating factor-1, Cyt c: cytochrome c, Cytp450: cytochrome P450, G2/M arrest: cell cycle “gap2” mitotic phase arrest, ASK-1: apoptosis signal-regulating kinase-1, PEG 2: polyethylene glycol 2, COX 2: cyclooxygenase 2, MAPK: mitogen-activated protein kinase, P38: mitogen-activated protein kinase protein 38, ERK: extracellular signal-regulated kinase, JNK: Jun N-terminal kinase, B-cells: B lymphocytes cells, TNF-alpha: Tumor necrosis factor - alpha, TGF-β: transforming growth factor beta, Ls: lipid hydro-peroxides, MMPs: matrix metalloproteinases, PGs: prostaglandins.

Inflammation is body’s normal response to illness and infection. When the immune system attacks the body’s own tissues, it results in inflammation. Rheumatoid arthritis (RA) is an example of an inflammatory disease that affects the joints (Zheng et al., 2015). The production of ROS in injured joints promotes inflammatory reactions. The cytokines generated play a role in the immunoregulatory and tissue damage processes developing clinical manifestations in RA (Direito et al., 2021). As human antioxidant defense systems are inefficient, exogenous antioxidants must be used to fight excess ROS (Sung et al., 2019; Direito et al., 2021). Polyphenols have the ability to regulate the inflammatory pathways of common arthropathies such as gout, osteoarthritis and RA. EGCG, quercetin, resveratrol, *p*-coumaric acid, luteolin, curcumin, kaempferol and apigenin are the most effective polyphenols against arthritis (Ahmed et al., 2006; Pragasam, 2012; Riegsecker et al., 2013; Abba et al., 2015; Chang et al., 2015; Daily et al., 2016; Aziz et al., 2018). Tea flavan-3-ols like EGCG are useful in RA (Jin et al., 2020). The effects of quercetin on disease severity and inflammation in women with RA showed considerably decreased early morning stiffness and discomfort and after-activity pain (Javadi et al., 2017). Kaempferol improved arthritis severity, cartilage degradation, inflammation and bone erosion in collagen induced arthritic (CIA) male DBA/J1 mice (Lee et al., 2018). Resveratrol shows its anti-rheumatoid arthritis properties with reduced RA patients’ swelling, tenderness, and disease activity by lowering the biochemical indicators of inflammation like MMP-3, IL-6, ESR, C-reactive protein, and undercarboxylated osteocalcin (Khojah et al., 2018; Meng et al., 2021). The protective effects of dietary polyphenols against cancer, infectious illness and inflammatory diseases are depicted in Figure 8.

## Pro-oxidative Effects of Dietary Polyphenols

Although much research has been focused on the antioxidant properties of plant-derived polyphenols against chronic diseases (neurodegenerative diseases, cardiovascular complications, cancer, diabetes, bacterial infections, and inflammations) as described above, they can also act as pro-oxidants in the biological systems (*in vivo*). The pro-oxidative action of polyphenols depends on certain factors such as their solubility characteristics, chelating behavior, metal-reducing potential etc. and the pH at the site of action (Babich et al., 2011). A variety of dietary polyphenols including gallic acid, ellagic acid, quercetin, myricetin, rutin, kaempferol, resveratrol, catechins, EGCG etc. exhibit such dual (antioxidant and pro-oxidative) roles. However, the anticancer, antiobesity and antimicrobial effects of green tea polyphenols (EGCG, ECG) are primarily because of their antioxidant activity, whereas the harmful toxic effects are due to their pro-oxidative effect (Ouyang et al., 2020). The pro-oxidant effect of EGCG (major ingredient of tea) is observed at considerably higher dose than that of the dose required for antioxidant action. The pro-oxidant capacity of tea polyphenols is such that they directly lead to the generation of ROS, and indirectly induces apoptosis and death of cancer cells (León-González et al., 2015). The grape seed extract exhibits *in vivo* pro-oxidant activity to an appreciable extent depending on dose, duration of administration, and other dietary components. As pro-oxidant molecules, polyphenols can exert cytotoxic effects against cancer cells by achieving toxic levels of ROS. Increased ROS level eventually induces DNA degradation in the presence of metal ions such as copper, which ultimately leads to cell death (D'Angelo et al., 2017). The pro-oxidant effect may also be associated with a pro-apoptotic function in various types of tumor cells (Khan et al., 2012). The pro-oxidative effect of resveratrol may counteract the tissue damage induced by oxidative stress (Chedea et al., 2021). Further, polyphenols including flavonoids and anthocyanins also play a potential pro-oxidant role and protects our body from severe cellular oxidative stress. For instance, red wine polyphenols may help modulate the antioxidant potential of erythrocytes, protecting them against oxidative stress (Chedea et al., 2020).

## Conclusion

Food phenolics are gaining importance in research as they have the potential to improve human health. Over 8,000 polyphenols have been reported from plants, and several hundreds of dietary polyphenols have been found in foods. Owing to their potent antioxidant capacity because of the presence of hydroxyl groups in their structures, polyphenols can effectively scavenge ROS and thus fight against OS induced pathological conditions or human diseases. Evidence from diverse *in vitro* studies discussed here supports that dietary sourced polyphenols plays a potential protective role in the prevention of neurodegenerative diseases, CVDs, diabetes, cancer, inflammation-related diseases, and infectious illness. However, prospective further research with adequate pre-clinical and clinical investigations could lead to the development dietary polyphenolic compounds as potent therapeutic candidates against various chronic human diseases.
